# Anxiety-like behavior in Rett syndrome: characteristics and assessment by anxiety scales

**DOI:** 10.1186/s11689-015-9127-4

**Published:** 2015-09-15

**Authors:** Katherine V. Barnes, Francesca R. Coughlin, Heather M. O’Leary, Natalie Bruck, Grace A. Bazin, Emily B. Beinecke, Alexandra C. Walco, Nicole G. Cantwell, Walter E. Kaufmann

**Affiliations:** Department of Neurology, Boston Children’s Hospital, Boston, MA 02115 USA; Harvard Medical School, 300 Longwood Avenue, Boston, MA 02115 USA

**Keywords:** Rett syndrome, Intellectual disabilities, Anxiety, Problematic behavior, Social avoidance

## Abstract

**Background:**

Rett syndrome (RTT) is a severe neurodevelopmental disorder characterized by regression of language and motor skills, cognitive impairment, and frequent seizures. Although the diagnostic criteria focus on communication, motor impairments, and hand stereotypies, behavioral abnormalities are a prevalent and disabling component of the RTT phenotype. Among these problematic behaviors, anxiety is a prominent symptom. While the introduction of the Rett Syndrome Behavioral Questionnaire (RSBQ) represented a major advancement in the field, no systematic characterization of anxious behavior using the RSBQ or other standardized measures has been reported.

**Methods:**

This study examined the profiles of anxious behavior in a sample of 74 girls with RTT, with a focus on identifying the instrument with the best psychometric properties in this population. The parent-rated RSBQ, Anxiety, Depression, and Mood Scale (ADAMS), and Aberrant Behavior Checklist-Community (ABC-C), two instruments previously employed in children with neurodevelopmental disorders, were analyzed in terms of score profiles, relationship with age and clinical severity, reliability, concurrent validity, and functional implications. The latter were determined by regression analyses with the Vineland Adaptive Behavior Scales-Second Edition (Vineland-II) and the Child Health Questionnaire (CHQ), a quality of life measure validated in RTT.

**Results:**

We found that scores on anxiety subscales were intermediate in range with respect to other behavioral constructs measured by the RSBQ, ADAMS, and ABC-C. Age did not affect scores, and severity of general anxiety was inversely correlated with clinical severity. We demonstrated that the internal consistency of the anxiety-related subscales were among the highest. Test-retest and intra-rater reliability was superior for the ADAMS subscales. Convergent and discriminant validity were measured by inter-scale correlations, which showed the best profile for the social anxiety subscales. Of these, only the ADAMS Social Avoidance showed correlation with quality of life.

**Conclusions:**

We conclude that anxiety-like behavior is a prominent component of RTT’s behavioral phenotype, which affects predominantly children with less severe neurologic impairment and has functional consequences. Based on available data on standardized instruments, the ADAMS and in particular its Social Avoidance subscale has the best psychometric properties and functional correlates that make it suitable for clinical and research applications.

## Background

Rett syndrome (RTT; OMIM 312750), the second most common cause of severe intellectual disability (ID) in females, is a neurodevelopmental disorder that affects 1 per 10,000 females by the age of 12 [1]. Over 95 % of individuals with RTT have mutations in methyl-CpG-binding protein (*MECP2)*, a gene on Xq28, which encodes a transcriptional regulator [2–4]. MeCP2 is a protein involved in synaptic development and maintenance [5, 6]. The diagnosis of RTT is clinical; approximately 98 % individuals who meet diagnostic criteria for typical RTT have a *MECP2* mutation, while about 80 % of those meeting criteria for atypical RTT carry a *MECP2* mutation [4, 7]. However, 3–5 % of individuals meeting diagnostic criteria for RTT do not have a *MECP2* mutation [4]. While the overall clinical presentation varies across individuals, some mutations have been associated with a more severe phenotype, including p.Arg106Trp, p.Thr158Met, p.Arg168X, p.Arg255X, p.Arg270X, and large deletions. However, other mutations have a relatively milder phenotype, such as p.Arg133Cys, p.Arg294X, p.Arg306Cys, and 3′ truncations [3, 8, 9]. Although RTT is not considered a progressive disorder, the clinical severity of most mutations increases with age [3].

The diagnosis of RTT is based on the gradual or sudden loss of previously acquired fine motor and expressive language skills, as well as the appearance of hand stereotypies and gait abnormalities [7]. The regression period can occur as early as 6 months or as late as 5 years but typically begins around 12 to 18 months of age [7, 10, 11]. Other common features of the disorder, which are considered supportive diagnostic criteria, include growth retardation, breathing disturbances when awake, scoliosis/kyphosis, and decreased response to pain [7]. Although only inappropriate laughing/screaming spells are considered a diagnostically supportive manifestation [7], behavioral abnormalities are prevalent and impairing in RTT [12–16]. Underscoring the close link between MeCP2 deficits and abnormal behavior, mouse models of Mecp2 deficiency recapitulate many of the features of the RTT phenotype including anxiety-like behaviors [17], impaired social behaviors [18], and stereotypic limb motions [19].

Earlier reports of abnormal behavior in humans with RTT focused on autistic features, which typically manifest during regression. In 1983, Hagberg and colleagues first described the disorder in the English literature as a ‘progressive syndrome of autism, dementia, ataxia, and loss of purposeful hand use in girls’ [20]. Since then, numerous studies have further evaluated the presence of autistic features in RTT and found that, along with the loss of expressive language and the emergence of repetitive hand movements, social withdrawal behaviors also contributed to the large proportion of individuals meeting Diagnostic and Statistical Manual of Mental Disorders (DSM)-IV criteria for pervasive developmental disorder not otherwise specified (PDD-NOS) [21–23]. Unlike these relatively well-described autistic features, significantly less is known about anxiety-like behaviors in RTT [13, 16].

Several studies report a high prevalence of anxiety and mood disturbances in RTT, such as self-abuse, screaming episodes, abrupt mood changes, and inconsolable crying [15, 16, 24–29]. Mount and colleagues made a major contribution to our understanding of aberrant behavior in RTT by developing the Rett Syndrome Behaviour Questionnaire (RSBQ), a rating scale specific to RTT that covers a wide range of problem behaviors [21]. Although the RSBQ has been validated against samples of females with intellectual disability of unknown cause, total scale scores are difficult to interpret since they are influenced by multiple items that are considered more neurologic than behavioral in nature, such as breathing abnormalities, gross motor function, and repetitive hand movements (e.g., Breathing Problems contribute 7.3 % variance vs. Fear/Anxiety 5.2 %) [21]. Furthermore, RSBQ subscale scores are not independent factors since they are highly correlated [16]. One of the first applications of the RSBQ to the characterization of anxiety and mood disturbances was the report in 2006 by Robertson et al. who examined genotype-behavioral phenotype correlations in a large cohort of RTT females (*n* = 135). Using the RSBQ’s General Mood and Fear/Anxiety subscales, the study reported that individuals with milder mutations (p.Arg133Cys, p.Arg306Cys, p.Arg294X) were more likely to report mood difficulties and/or signs of fear/anxiety. The association was inverse in those with more severe mutations, who were less likely to report mood disturbances (p.Thr158Met) or fear/anxiety (p.Arg168X) [25]. A recent update to these data reports that individuals with p.Arg133Cys, p.Arg294X, and large deletions are more likely to experience mood problems and that those with p.Thr158Met and p.Arg168X mutations had reduced likelihood of anxiety [12].

Although literature on the subject is lacking, clinical experience in RTT demonstrates that anxiety symptoms may be severe enough to require treatment with psychoactive medications, such as, the selective serotonin reuptake inhibitor (SSRI), escitalopram. In a phase 1 trial (NCT01253317) of insulin-like growth factor (IGF-1) involving nine subjects with classic RTT, the RSBQ and the Anxiety, Depression, and Mood Scale (ADAMS), a recently developed instrument for assessing these domains in individuals with intellectual disability [30–32], were administered in a pilot evaluation of IGF-1 efficacy. This 20-week open-label treatment study showed modest although consistent improvements in both the RSBQ Fear/Anxiety and the ADAMS Social Avoidance subscales [33]. These findings were supported by the partial or complete reversal of right-sided alpha band frontal EEG asymmetry, which has been used as a biomarker of anxiety or depression [34], in five of the six subjects with available EEG data [33]. This work suggests that the RSBQ and ADAMS anxiety- and mood-related subscales have construct validity and could be used as outcome measures in clinical trials. The IGF-1 phase 1 trial and its preclinical counterpart using Mecp2 deficient mice [35] also support the notion that anxiety-like behaviors are a promising therapeutic target in RTT.

The literature reviewed in the preceding sections emphasizes the importance of further elucidating the anxiety profile in RTT and underscores the need for characterizing the psychometric properties of existing behavioral rating scales such as the RSBQ and the ADAMS. Evaluation of behavior in RTT is complicated by the characteristic marked communication and motor impairments, including psychomotor slowing, displayed by most affected individuals. This leads to a greater reliance on parent-based observations since, for ecological and clinical validity, data need to be collected across different settings and for longer periods of time. The present study examines the profiles of anxious behavior in a sample of 74 girls with RTT, with a focus on further delineating the range of manifestations and their relationship with other abnormal behaviors and clinically relevant parameters. We also intended to identify the instrument with the best psychometric properties in this population. For this purpose, we studied the anxiety-oriented subscales of the RSBQ and the ADAMS and contrasted them with equivalent subscales of the Aberrant Behavior Checklist-Community (ABC-C) [36], the most widely used instrument for assessing abnormal behavior in individuals with intellectual disability [37–39], and with other measures of abnormal behavior, functioning, and quality of life.

## Methods

### Participants

Data was gathered in 74 girls, ages ranging from 2 to 11 years (*M* = 5.35; *SD* = 2.36), with typical RTT and a confirmed *MECP2* mutation. The majority of participants (*n* = 57) were pre-screening candidates for the phase 2 IGF-1 trial (NCT01777542) currently underway at the Boston Children’s Hospital. Surveys for the remaining 17 subjects were collected as a part of a separate study at the BCH aimed at characterizing visual evoked potentials in young children with RTT and from the phase 1 IGF-1 trial (pre-treatment). For the three studies mentioned above, parents are asked to complete the RSBQ, ADAMS, and a detailed developmental and medical history questionnaire and to provide copies of their child’s MECP2 mutation results and growth charts and pediatric medical records from their first 3 years of life. If necessary, ambiguities are clarified through a phone interview with parents and additional records are requested from the child’s school or early intervention service provider. Using these records, we determine if the child meets criteria for typical or atypical RTT and define the child’s developmental staging in terms of their regression status. For the purposes of this analysis, any child that had experienced skill loss within the 6 months preceding the data of assessment were determined as being in the active stage of regression (Hagberg stage 2). All children included in this work were classified as having typical Rett syndrome, and nine of the girls between the ages of 2 and 4 years were determined to be in Hagberg stage 2. The vast majority of the individuals (*n* = 65) were post-regression (Hagberg stage 3). The aforementioned studies and their respective data collection procedures were approved by the Institutional Review Board at the Boston Children’s Hospital.

### Measures

#### Rett Syndrome Behavior Questionnaire (RSBQ)

The RSBQ is an informant/parent-completed measure of abnormal behaviors typically observed in individuals with RTT, which is completed by a parent or caregiver [24]. Each item, grouped into eight domains/subscales (i.e., General Mood, Breathing Problems, Body Rocking and Expressionless Face, Hand Behaviors, Repetitive Face Movements, Night-time Behaviors, Fear/Anxiety, and Walking/Standing), is scored on a Likert scale of 0–2, according to how well the item describes the individual’s behavior [24]. For the analyses of anxious behaviors, we only included the Fear/Anxiety subscale. Nonetheless, for data interpretation, we also used the scores of the General Mood and other subscales.

#### Anxiety Depression and Mood Scale (ADAMS)

\The ADAMS is another parent/caregiver-completed measure and consists of 28 items, grouped into five subscales (Manic/Hyperactive Behavior, Depressed Mood, Social Avoidance, General Anxiety, Obsessive Behavior), and scored on a four-point Likert scale that combines frequency and severity ratings [31]. It is the only empirically derived instrument for assessing affective disorders in ID. It is not restricted by the individual’s handicaps, such as communicative level, and it does not rely on the DSM framework [30, 31]. The ADAMS was normed in 265 individuals and validated in 129 psychiatric patients with ID [31], and it has been used to characterize anxiety disorders in fragile X syndrome [30]. Previous research examining the correlations between the ADAMS and ABC-C subscales demonstrated good convergent and discriminant validities [32]. Our analyses focused on the Social Avoidance and General Anxiety subscales; however, other subscales were used for data interpretation.

#### Aberrant Behavior Checklist-Community (ABC-C)

The ABC-C is an informant-based behavior rating scale originally designed for measuring drug and other treatment effects in individuals with ID [36]. Its 58 items cover a wide range of behaviors, scored between 0–3, and grouped in five empirically derived subscales (Irritability, Lethargy/Social Withdrawal, Inappropriate Speech, Hyperactivity, and Stereotypic Behavior). A revision of the ABC-C for fragile X syndrome generated another subscale, derived from Lethargy/Social Withdrawal items, with empirical observation basis termed Social Avoidance [anxious behaviors] that has also been applied to studies of autism spectrum disorder [37, 38]. The ABC-C is the most widely employed measure of abnormal behavior in ID and autism spectrum disorder, with applications ranging from characterizing behavioral phenotype to measuring response to interventions [37–40]. Our analyses focused on the two anxiety-related subscales, which mainly cover social anxiety-related behaviors: Lethargy/Social Withdrawal and Social Avoidance. As for the RSBQ and ADAMS, we contrasted scores on these subscales with the other components of the ABC-C.

#### Clinical Severity Scale (CSS)

For the past ten years, the CSS has been used to assess overall clinical severity in the longitudinal Rett Syndrome Natural History study (U54 HD061222) involving over 1100 children and adults with RTT and/or *MECP2* mutations [3, 4, 8, 41]. The CSS includes 13 items specific to the RTT phenotype, both historical and measuring current clinical severity: age of onset of regression, somatic growth, head growth, independent sitting, ambulation (independent or assisted), hand use, scoliosis, language, non-verbal communication, respiratory dysfunction, autonomic symptoms, onset of stereotypies, and seizures. Based on the severity or degree of abnormality, each item is scored on a 0–5 scale. In this study, we only analyzed total CSS scores.

#### Vineland Adaptive Behavior Scales-II (Vineland-II)

The Vineland-II, survey interview form, was designed to assess handicapped and non-handicapped persons in their personal and social functioning [42]. The survey interview form is administered to a parent or caregiver using a semi-structured interview format, organized into four domains: Communication, Daily Living Skills, Socialization, and Motor Skills. A standard score and age equivalency are calculated for each domain, as well as a total score termed Adaptive Behavior Composite. The VABS-II has been validated in children and adults with ID and used in populations such as autism spectrum disorder, fragile X syndrome, Down syndrome, Angelman syndrome, and RTT [13, 43–46]. In the present study, we only analyzed Vineland-II domain standard scores.

#### Child Health Questionnaire (CHQ)

The Child Health Questionnaire-Parent Form 50 (CHQ-PF50) is a quality of life scale of 50 items which was designed to measure the physical and psychosocial well-being of children [47, 48]. The CHQ includes 14 different subscales that can be combined to derive overall Physical and Psychosocial Summary Scores. The CHQ was normed in children ages 5 to 10 years old and has been used extensively in children with chronic diseases and neurodevelopmental disorders, including RTT [47, 49, 50]. In accordance with previous reports, we focused on the Physical and Psychosocial Summary Scores to allow for comparisons.

#### Statistical analyses

In addition to performing descriptive and data distribution analyses, we conducted a variety of comparative and other specialized statistical analyses aiming at delineating the relationship between the abovementioned measures and characterizing the psychometric properties of the anxiety scales. Specific tests are described below in each “Results” section. The overall analytical strategy can be summarized as follows: (1) characterizing the RTT cohort’s behavioral profile by the three behavioral rating scales, with emphasis on determining the relative severity of anxiety-like behaviors with respect to other problem behaviors; (2) determining the score distribution of the anxiety-related subscales; (3) examining the relationship between anxiety scale scores and basic parameters such as age and clinical severity; (4) evaluating the reliability of anxiety scales, specifically internal consistency by the Cronbach’s alpha, and test-retest and intra-rater reliabilities; (5) determining convergent and discriminating validity of the anxiety subscales, measured as the relationship between themselves and with subscales measuring other constructs; and (6) determining the functional significance of anxiety scale scores, by evaluating their relationship with the Vineland-II and the CHQ. Due to data distribution, we used non-parametric regression analyses and *t* tests. One- or two-sided/tailed tests were used, where appropriate, as well as Bonferroni corrections for multiple comparisons.

## Results

### General behavior profile in RTT

On the RSBQ, our cohort showed subscale median scores approaching the measure’s median (based on the potential score range of each subscale) with exception of Hand Behaviors, a subscale that reflects the characteristic hand stereotypy of RTT (Table 1). Our cohort’s median score on the subscale representing anxiety (Fear/Anxiety) was slightly above the measure’s median. Scores on the ABC-C subscales were relatively low and, similar to the RSBQ, only the Stereotypy subscale showed a median slightly above the measure’s median. Scores on the ADAMS were relatively low but comparatively higher than those on the ABC-C, with only two scales, Manic/Hyperactive Behavior and General Anxiety, with medians approaching the measure’s median and values in the upper range. In general, distribution of scores for the anxiety-related subscales was comparable to that of mood subscales and intermediate between those for stereotypic behavior (upper end) and disruptive behavior (lower end).

**Table 1:** Descriptive statistics

CSS	Number	Min.	Max.	Skewness	SE	Kurtosis	SE	25th	50th	75th	85th	95th	Items	α
Total	51	5	32	−0.1	0.3	−0.3	0.7	16.00	20.00	25.00	27.00	30.40	13	–
RSBQ														
General Mood	74	0	16	0.5	0.3	−0.6	0.6	4.00	6.00	9.50	13.00	15.30	8	0.88
Body Rocking	74	0	14	0.6	0.3	0.1	0.6	4.00	6.00	8.00	9.00	12.30	7	0.67
Hand Behaviors	74	3	12	−0.5	0.3	−0.5	0.6	6.00	8.00	10.00	10.00	12.00	6	0.63
*Fear/Anxiety*	*74*	*0*	*8*	*−0.1*	*0.3*	*−0.7*	*0.6*	*3.00*	*5.00*	*6.00*	*7.00*	*8.00*	*4*	*0.73*
Breathing Problems	72	0	10	0.3	0.3	−0.9	0.6	2.00	4.50	7.00	8.00	10.00	5	0.76
Repetitive Face Movements	74	0	8	0.0	0.3	−0.8	0.6	2.00	4.00	5.00	6.00	7.00	4	0.53
Night-time Behaviors	72	0	6	0.9	0.3	−0.1	0.6	0.00	1.00	3.00	4.00	5.30	3	0.75
Walking/Standing	73	0	4	−0.1	0.3	−1.3	0.6	1.00	2.00	3.00	4.00	4.00	2	0.62
Total													45	0.88
ADAMS														
Manic	74	0	14	0.3	0.3	−0.8	0.6	3.00	6.00	9.00	10.90	13.00	5	0.77
Depressed	74	0	19	1.3	0.3	2.6	0.6	1.00	4.00	6.00	8.00	11.40	7	0.76
*Social Avoidance*	*74*	*0*	*16*	*1.1*	*0.3*	*1.3*	*0.6*	*2.50*	*4.00*	*7.00*	*8.00*	*15.30*	*7*	*0.80*
*General Anxiety*	*74*	*0*	*20*	*1.0*	*0.3*	*0.4*	*0.6*	*3.00*	*5.00*	*9.00*	*12.20*	*18.40*	*7*	*0.91*
Obsessive Behavior	73	0	7	0.7	0.3	−0.4	0.6	1.00	2.00	4.75	6.00	7.35	3	0.60
Total													28	0.92
ABC-C														
Irritability	47	0	29	1.3	0.4	1.1	0.7	3.75	7.00	12.00	17.95	27.60	15	0.89
*Lethargy/Social Withdrawal*	*47*	*0*	*22*	*0.7*	*0.4*	*−0.4*	*0.7*	*4.00*	*8.00*	*13.25*	*16.00*	*21.30*	*16*	*0.78*
Stereotypy	47	1	18	−0.2	0.4	0.9	0.7	9.00	11.00	13.25	14.95	17.30	7	0.68
Hyperactivity	47	0	36	1.4	0.4	1.6	0.7	5.00	8.00	14.50	22.00	34.60	16	0.93
Inappropriate Speech	47	0	10	4.4	0.4	23.0	0.7	0.00	0.00	0.00	1.95	3.00	4	0.86
*Social Avoidance*	*47*	*0*	*10*	*1.6*	*0.4*	*3.0*	*0.7*	*0.00*	*0.50*	*3.25*	*4.00*	*5.65*	*4*	*0.86*
Total													58	0.94

There were no differences in the mean/median scores in any of the five anxiety-related scales (in italics), when the nine subjects in the regression stage were compared with the 65 individuals post-regression. Interpretative guidelines for internal consistency by Cortina [53]: 0.90 or above = excellent; 0.80–0.89 = good; 0.70–0.79 = fair; below 0.70 = unacceptable. In italics are descriptive statistics for the five anxiety-related measures

### Internal structure of anxiety scales in RTT

We examined the distribution of scores for each of the subscales using percentile ranks for the scores (Table 1). The percentile rank of a measure’s score is the percentage of scores at the same level or below it [51]. Percentile ranks are useful in demonstrating score distribution beyond the median, particularly the level of upper range skewing. In terms of the RSBQ, the Fear/Anxiety subscale showed a comparable percentile rank distribution to other subscales, whether they measured mood abnormalities or other behaviors. The ADAMS Social Avoidance displayed a wide range of scores between the median and percentile 85 comparable to other ADAMS subscales but different from the General Anxiety subscale, which had a more even distribution of upper range scores. The two social anxiety-related ABC-C subscales showed a similar pattern of score distribution to the ADAMS Social Avoidance, which was comparable to other ABC-C subscales with exception of the Stereotypy (probably reflecting the relatively high scores on this subscale in the entire RTT cohort). To our knowledge, no percentile rank distribution has been reported for the RSBQ and ABC-C score distribution is quite variable between and within genetic disorders depending on the proportion of individuals with severe autistic behavior as we have reported for fragile X syndrome [37, 52] and Down syndrome [39]. For the ADAMS, the percentile ranks in our RTT cohort were comparable to those reported by Esbensen and colleagues in 2003 [31] in a large population of individuals with intellectual disability and by Rojahn and colleagues in 2011 [32]. Skewness and kurtosis values were variable among anxiety-related subscales, with the lowest skewness corresponding to the RSBQ Fear/Anxiety and lowest kurtosis to the ABC-C Social Avoidance (Table 1). In general, the values of these distribution parameters were not substantially different from other construct subscales and comparable or better than those reported by Rojahn et al. [32].

### Anxiety scales, age, and clinical severity in RTT

#### Relationship with age

We assessed the effect of age on anxiety scale scores in two ways (Table 2). First, we correlated age as a continuous variable using Spearman regression analyses and adjusting for multiple comparisons by Bonferroni corrections for the five anxiety-related measures (corrected *p* = 0.010) (Table 3). Only the RSBQ Fear/Anxiety demonstrated a near trend level direct relationship. Because this suggested that older children are more likely to demonstrate more severe general anxiety, we also evaluated the effect of age by dividing the cohort into two groups below (*n* = 45) and equal or above (*n* = 29) 6 years. This cutoff was selected because by this age, most children with RTT have finished their regression and have entered a more stable stage of the disease, from the developmental skill viewpoint [4, 7]. However, no age group differences were found for the RSBQ Fear/Anxiety or any of the other four anxiety-related subscales (Table 3). To further probe the question of age, we divided our cohort into four age groups (2–4 years, 4–6 years, 6–8 years, and 8–10+ years) and conducted Welch-Aspin *t* tests (unequal variances) comparing the mean scores of the four groups on all five anxiety-related subscales and found no significant differences (data not shown). To address concerns that the nine subjects still in the regression stage of the disorder could potentially influence our results, we conducted comparative analyses using non-parametric *t* tests between subjects in stage 2 and those in stage 3 and found there were no significant differences between the two groups in any of the five anxiety-related subscales. It is important to note that data for all nine subjects still in regression were only available for the RSBQ and ADAMS. We also compared CSS scores (see next section) between these two groups, based on data from four individuals in stage 2 and 46 subjects in stage 3. While no significant differences were observed among anxiety subscales, the CSS scores of individuals in the post-regression stage were significantly higher.

**Table 2:** Age grouping

Ages (years)	Number
2–4^a^	26
4–6	20
6–8	14
8–11	15
Total	75

^a^Nine subjects had experienced skill loss within 6 months prior to assessment

**Table 3:** Anxiety scale relationship with age and clinical severity

		Age				CSS	
	*N*	Spearman’s rho	*p* value	^a^Mann-Whitney *U*	^a^Asymp. sig (two-tailed)	Spearman’s rho	*p* value
CSS	51	0.101	0.241	249.500	0.348	–	–
RSBQ							
Fear/Anxiety	74	0.182	0.060	575.500	0.248	−0.236	**0.048**
ADAMS							
Social Avoidance	74	−0.079	0.251	435.000	0.391	−0.220	0.060
General Anxiety	73	0.120	0.156	563.000	0.446	−0.331*	**0.009**
ABC-C							
Lethargy/Social Withdrawal	47	0.010	0.474	218.500	0.623	−0.138	0.177
Social Avoidance	47	0.006	0.484	223.000	0.676	−0.150	0.157

In bold, significant and trend level *p* values

*Adjusted for multiple [5] comparisons (excluding CSS) with Bonferroni corrections for the five anxiety-related measures, adjusted *p* = 0.01

^a^Age grouping <6 years (*n* = 45), >6 years (*n* = 29)

#### Relationship with clinical severity

We evaluated the relationship between anxiety scales and clinical severity in two ways. First, by regression analyses with the CSS, a global measure of severity that incorporates multiple aspects of the disorder except does not include behavioral features. Using Spearman regression and adjusting again for multiple comparisons, with a corrected alpha of 0.01, we found that the ADAMS General Anxiety was inversely correlated with the CSS (*p* = 0.009). The RSBQ Fear/Anxiety was also inversely correlated with the CSS at the trend level (*p* = 0.048), and the ADAMS Social Avoidance approached an inverse trend (*p* = 0.060). Neither ABC-C subscale was related to the CSS (Table 3). We complemented this first approach by comparing scores of those subjects with mutations considered milder or more severe, in keeping with previous studies of other phenotypical aspects of RTT [3, 4, 9]. The “milder” mutation group included p.Arg133Cys, p.Arg294X, p.Arg306Cys, 3′ truncations, and other point mutations, while the “more severe” mutation group included p.Arg106Trp, p.Thr158Met, p.Arg168X, p.Arg255X, p.Arg270X, splice sites, and large deletions and insertions. Mann-Whitney *t* tests corrected for the five comparisons supported the abovementioned findings, in that girls with “milder” mutations were found to have higher scores on the ADAMS General Anxiety (*p* = 0.013) (Table 4).

**Table 4:** Anxiety scale relationship with *MECP2* mutation severity

		Mild (*n* = 26)		Severe (*n* = 46)		Mutation differences	
	*N*	Median	SD	Median	SD	^a^Mann-Whitney *U*	^a^Asymp. sig (two-tailed)
CSS	51	18.00	5.47	22.00	5.54	175.00*	**0.009**
RSBQ							
Fear/anxiety	72	5.00	2.11	4.00	2.01	504.50	0.267
ADAMS							
Social avoidance	72	4.00	3.29	4.00	3.69	515.50	0.330
General anxiety	71	7.00	4.32	5.00	4.80	377.00	**0.013**
ABC-C							
Lethargy/social withdrawal	47	9.50	6.46	6.50	4.47	208.00	0.207
Social avoidance	47	1.50	2.79	0.00	1.34	208.50	0.179

In bold, significant and trend-level *p* values

*Adjusted for multiple [5] comparisons (excluding CSS) with Bonferroni corrections for the five anxiety-related measures, adjusted *p* = 0.01

^a^Grouping mild vs. severe *MECP2* mutation

For reference, we also evaluated the relationship between age and both CSS and mutation category, revealing that subjects in the more severe mutation category had higher CSS scores (*p* = 0.009) (Table 4).

### Reliability of anxiety scales in RTT

#### Internal consistency

We measured the internal consistency of all subscales of the RSBQ, ADAMS, and ABC-C in our RTT cohort using Cronbach’s alpha, which calculates the level of correlation between a given item on a subscale with the remaining items on the same subscale (Table 1). Interpretative guidelines for internal consistency [53] state an alpha coefficient of 0.90 or above is excellent; 0.80–0.89, good; 0.70–0.79, fair; and below 0.70, unacceptable. Here, we comment only on the anxiety-related scales. For the RSBQ, the alpha coefficient for Fear/anxiety (0.73) was comparable to the one reported by Mount et al. in their 2002 publication (0.66–0.74) [24]. Alpha coefficients in our RTT cohort for the ADAMS General Anxiety (0.91) and Social Avoidance (0.80) were comparable to findings reported for ID by Esbensen and colleagues [31] and by Rojahn et al. [32]. We observed similar patterns in the ABC-C with alpha coefficients of 0.78 for Lethargy/Social Withdrawal and 0.86 for Social Avoidance. Thus, alpha coefficients for the anxiety subscales under investigation ranged from fair (0.70–0.79) to excellent (0.90 and above).

#### Test-retest reliability

Table 5 depicts our evaluation of test-retest reliability (*n* = 34) for the anxiety subscales of the RSBQ and ADAMS using intraclass correlation coefficients (ICCs) with a one-way random effects model (ICC average measures coefficient) [54, 55]. Guidelines for the interpretation of inter- and intra-examiner levels of agreement indicate that when the reliability coefficient (in our case ICC) is 0.75 or greater, the level of clinical significance is excellent, from 0.60 to 0.74, good; 0.40 to 0.59, fair; and below 0.40, poor. The RSBQ Fear/Anxiety subscale exhibited good retest reliability with an ICC of 0.72, which is lower than previous findings [24]. The ADAMS Social Avoidance and General Anxiety subscales both had excellent retest reliability at 0.80 and 0.81, respectively; both of which are comparable to previous reports [31].

**Table 5:** Test-retest reliability and intra-rater reliability for anxiety subscales

	Number	Retest	95 % CI	Intra-rater	95 % CI
RSBQ					
General Mood	34	0.86	0.72–0.93	0.75	0.56–0.87
Body Rocking and Expressionless Face	34	0.90	0.80–0.95	0.82	0.67–0.90
Hand Behaviors	34	0.83	0.67–0.92	0.71	0.49–84
*Fear/Anxiety*	*34*	*0.72*	*0.45–0.86*	*0.57*	*0.29–0.76*
Breathing Problems	34	0.91	0.82–0.95	0.83	0.69–0.91
Repetitive Face Movements	34	0.78	0.56–0.88	0.64	0.38–0.79
Night-time Behaviors	34	0.94	0.88–97	0.89	0.79–0.94
Walking/Standing	34	0.74	0.49–0.87	0.59	0.32–0.77
Mean		0.84	–	0.73	–
ADAMS					
Manic/Hyperactive Behavior	34	0.77	0.54–0.88	0.62	0.37–0.79
Depressed Mood	33	0.72	0.44–0.86	0.57	0.29–0.76
*Social Avoidance*	*34*	*0.80*	*0.59–0.89*	*0.66*	*0.42–81*
*General Anxiety*	*33*	*0.81*	*0.61–0.91*	*0.68*	*0.45–0.83*
Obsessive/Compulsive Behavior	33	0.80	0.59–0.89	0.66	0.42–0.82
Mean		0.78	–	0.64	–

In italics are ICCs for anxiety-related subscales and 95 % confidence interval (CI)

#### Intra-rater reliability

We also evaluated intra-rater reliability (Table 5) on the anxiety subscales of the RSBQ and ADAMS using intraclass correlation coefficients (ICCs) with a one-way random effects model (ICC single measure coefficient) [55]. Intra-rater reliability for the RSBQ Fear/Anxiety was fair (0.57). To the authors’ knowledge, no reports on the RSBQ’s inter- or intra-rater reliability have been published. The intra-rater reliability for the ADAMS Social Avoidance and the General Anxiety subscales was considered good with a coefficient of 0.66 and 0.68, respectively. These findings are higher than those reported by Esbensen et al. [31] for intellectual disability, particularly so for the General Anxiety subscale. Due to differences in study design, we were unable to calculate ICCs for subscales of the ABC-C.

### Convergent and discriminant validity of anxiety scales in RTT

We estimated convergent validity by measuring the level of agreement between the five anxiety-related subscales. Discriminant validity was determined by the level of disagreement between these five subscales and subscales of the RSBQ and ABC-C measuring a different construct (e.g., RSBQ Breathing Problems, ABC-C Stereotypy). Because of the close relationship between anxiety and mood, we hypothesized that there would be an intermediate level of agreement between subscales measuring these two constructs. Therefore, mood-related subscales would not be informative in terms of either convergent or discriminant validity. These correlations were performed by one-tailed Spearman regression analyses since we expect all relationships to be in the same direction. As shown in Table 6, which includes the five anxiety-related subscales as well as one of the mood subscales (RSBQ General Mood) and another subscale partially reflecting mood behaviors (ABC-C Irritability), there were strong correlations between the three social anxiety subscales (ADAMS Social Avoidance, ABC-C Lethargy/Social Withdrawal, ABC-C Social Avoidance) and between the two general anxiety subscales (RSBQ Fear/Anxiety, ADAMS General Anxiety). While the ADAMS Social Avoidance was also correlated with the ADAMS General Anxiety and the RSBQ Fear/Anxiety, these relationships were weaker. On the other hand, the ABC-C social subscales were borderline or not correlated with the two general anxiety subscales. Table 6 also demonstrates that anxiety measures correlated more variably with mood subscales. In terms of discriminant validity, the two general anxiety subscales were also correlated with subscales measuring other constructs (only correlations with ABC-C Irritability shown on Table 6; other examples include RSBQ Body Rocking and ABC-C Hyperactivity). In contrast, with exception of subscales representing stereotypic behavior, the social anxiety scales correlated only with the mood or anxiety measures.

**Table 6:** Convergent and discriminant validity of anxiety subscales

	RSBQ General Mood	RSBQ Fear/Anxiety	ADAMS Social Avoidance	ADAMS General Anxiety	ABC Irritability	ABC Lethargy/Social Withdrawal	ABC Social Avoidance
RSBQ General Mood	1.000	0.430^b^	0.362^b^	0.347^b^	0.665^b^	0.434^b^	0.356^b^
*N*	74	74	74	73	47	47	47
RSBQ Fear/Anxiety			0.296^b^	0.626^b^	0.331^a^	0.160	0.218
*N*			74	73	47	47	47
ADAMS Social Avoidance				0.411^b^	0.277^a^	0.588^b^	0.613^b^
*N*				73	47	47	47
ADAMS General Anxiety					0.348^b^	0.251^a^	0.187
*N*					46	46	46
ABC Irritability						0.385^b^	0.240
*N*						47	47
ABC Lethargy/Social Withdrawal							0.690^b^
*N*							47
ABC Social Avoidance							1.000
*N*							47

^a^Correlation is significant at the 0.05 level (one-tailed)

^b^Correlation is significant at the 0.01 level (one-tailed)

### Functional correlates of anxiety scales in RTT

#### Anxiety and adaptive behavior

Using two-tailed Spearman regression analyses, we assessed how anxiety may influence adaptive behavior by correlating the five anxiety-related subscales with the Vineland-II (Table 7). Since the Vineland-II has four subdomains/scales, we implemented Bonferroni corrections to adjust for multiple comparisons (adjusted *p* = 0.0125). We only observed weak inverse relationships between the ADAMS Social Avoidance and the Vineland-II Communication (*p* = 0.12), the adaptive behavior domain more closely related to overall cognitive function, and the ABC Lethargy/Social Withdrawal and the Vineland-II Daily Living (*p* = 0.10). A stronger direct correlation was found between the ADAMS General Anxiety and the Vineland-II Motor (*p* = 0.04). Similar relationships with motor function were found for the RSBQ General Mood and the ABC-C Irritability. These findings suggest that RTT children with more severe social avoidance have slightly worse adaptive behavior skills and that those with more severe general anxiety or mood abnormalities have better motor skills.

**Table 7:** Correlations between anxiety subscales and Vineland Adaptive Behavior Scales (VABS-II) standard scores in RTT

		RSBQ General Mood	RSBQ Fear/Anxiety	ADAMS Social Avoidance	ADAMS General Anxiety	ABC Irritability	ABC Lethargy/Social Withdrawal	ABC Social Avoidance
Communication	Corr. coeff.	−0.075	0.030	−0.242	0.087	−0.017	−0.133	−0.151
	Sig.	0.637	0.853	0.122	0.585	0.916	0.401	0.340
	*N*	42	42	42	42	42	42	42
Daily Living Skills	Corr. coeff.	0.011	0.086	−0.158	−0.020	−0.124	−0.255	−0.223
	Sig.	0.944	0.589	0.319	0.897	0.436	0.104	0.156
	*N*	42	42	42	42	42	42	42
Socialization	Corr. coeff.	−0.205	−0.182	−0.107	−0.141	−0.186	−0.189	−0.207
	Sig.	0.193	0.248	0.499	0.374	0.239	0.231	0.189
	*N*	42	42	42	42	42	42	42
Motor Skills	Corr. coeff.	0.344*	0.218	0.074	0.312*	0.385**	0.087	0.140
	Sig.	0.026	0.166	0.642	0.044	0.012	0.582	0.376
	*N*	42	42	42	42	42	42	42

Two-tailed Spearman’s rho, adjusted for multiple comparisons with Bonferroni corrections for the four Vineland-II domains (*p* = 0.05/4, adjusted alpha 0.0125).

*p = 0.0125–0.05 (trend); **p < 0.0125 (significant)

#### Anxiety and quality of life

Using the same analytical strategy we employed for adaptive behavior, we also evaluated the impact of anxiety on quality of life in our RTT cohort (Table 8). We analyzed the two general scores of the CHQ, a quality of life measured validated for RTT [50]: the CHQ Physical Summary Score and the CHQ Psychosocial Summary Score. Considering an adjusted significance alpha of 0.025, only the ADAMS Social Avoidance was inversely correlated with the CHQ Psychosocial Scale at a trend level (*p* = 0.041). Including again other non-anxiety subscales in the analyses, we also found that the ABC-C Irritability subscale was strongly inversely correlated with the CHQ Psychosocial Scale (*p* = 0.002). These findings demonstrate that more severe selective anxiety and mood/disruptive behavior symptoms lead to a worse quality of life, which is in line with similar results using the ABC-C in fragile X syndrome [56].

**Table 8:** Correlations between anxiety subscales and quality of life in RTT

		RSBQ General Mood	RSBQ Fear/Anxiety	ADAMS Social Avoidance	ADAMS General Anxiety	ABC Irritability	ABC Lethargy/Social Withdrawal	ABC Social Avoidance
CHQ Physical Summary Measure	Corr. coeff.	−0.165	0.055	−0.031	0.068	−0.145	−0.015	0.213
	Sig.	0.328	0.744	0.856	0.688	0.422	0.936	0.234
	*N*	37	37	37	37	33	33	33
CHQ Psychosocial Summary Measure	Corr. coeff.	−0.310	−0.193	−0.338*	−0.298	−0.509**	−0.090	−0.060
	Sig.	0.062	0.252	0.041	0.073	0.002	0.620	0.739
	*N*	37	37	37	37	33	33	33

Two-tailed Spearman’s rho, adjusted for multiple comparisons with Bonferroni corrections for the two CHQ summary scores (*p* = 0.05/2, adjusted alpha: 0.025)

*p = 0.025–0.05 (trend); **p < 0.025 (significant)

## Discussion

Behavioral abnormalities are an important component of the phenotype of RTT [13, 16, 24, 33]. Nonetheless, they are not included in the diagnostic criteria of the disorder and their characterization is still limited. Until recently, most studies had focused on the autistic features of RTT. The incorporation of the RSBQ to the battery of instruments for evaluating individuals with RTT in 2002 brought attention to the frequency and severity of anxiety-like and other problem behaviors in RTT. Nevertheless, no systematic evaluation of anxious behaviors in RTT has been conducted. Taking into consideration the marked communication and motor impairments in the disorder, it is critical to delineate behaviors evaluated by standardized instruments in suitable RTT cohorts and not to assume that these measures are adequate for the disorder. Here, we report on a comprehensive assessment of the anxiety-related subscales of the RSBQ, ADAMS, and ABC-C. We determined both the profiles of anxious behaviors in RTT and the psychometric properties of the abovementioned instruments. We found that anxiety-like behaviors were comparable in severity to other behavioral domains such as mood abnormalities and disruptive behavior, but less pervasive than stereotypic behaviors. We also determined that, although relatively constant during childhood, anxious behaviors were more prominent in RTT children with milder neurologic impairment. Finally, while reliability measures were satisfactory for all five anxiety-related subscales, those evaluating the social domain showed better concurrent (convergent and discriminant) validity and greater functional implications. Based on the available data, the ADAMS Social Avoidance subscale displayed the best overall psychometric profile.

The RSBQ, ADAMS, and ABC-C profiles of our RTT cohort demonstrate a wide range of scores, with a relative prominence of stereotypic and repetitive behaviors that are captured mainly by the RSBQ Hand Behaviors and ABC-C Stereotypy subscales. Therefore, these scores are driven by the persistent hand stereotypies which are a cardinal feature of the disorder [7, 13]. Although some children with RTT display maximum scores on the anxiety-related subscales, the median scores for these measures were relatively low and predominantly below the range midpoint. Distribution of scores for the anxiety-related subscales was comparable to that of the mood subscales and intermediate between those for the stereotypic behavior and disruptive behavior subscales. Percentile ranks and skewness and kurtosis measures complemented the analyses of score distributions, revealing that, in general terms, anxiety-related subscales were not substantially different from subscales measuring other behavioral domains. We could conclude that anxious behaviors are relatively moderate in severity in RTT, and that general anxiety tends to be slightly more severe than social anxiety in this population. To determine how representative our cohort was, we compared its RSBQ profile with those published by Mount and colleagues (*n* = 139) and more recently by Cianfaglione et al. (*n* = 91). With the exception of the General Mood in the 2002 study, raw scores reported here are comparable to the two previous studies. Since no data have been published on ADAMS and ABC-C profiles in children with RTT (i.e., only one ABC-C study in adults with RTT has been published [57]), it is difficult to evaluate the representativeness of our cohort for these scales. Nevertheless, the ADAMS percentile ranks in this study were comparable to those of a large cohort with ID [31]. We found that age did not influence scores on anxiety-related subscales, which makes these measures suitable for a large segment of the RTT population. Since most of our cohort was younger than 8 years, and we included 9 subjects still in regression whose scores were comparable to the 65 in post-regression, we are confident that the three measures can be used in young children. We found that clinical severity only influenced the general anxiety measures, with neurologically less affected individuals having higher scores on the RSBQ Fear/Anxiety and ADAMS General Anxiety. This pattern was also found for other subscales, notably the ABC-C Irritability which represents disruptive behavior (data not shown). The fact that less impaired children with RTT present with more severe behavioral problems is not surprising since this has been the clinical impression for years. Whether these relationships are related to the child’s higher cognition and awareness of the environment, or because they are easier to assess due to better communication and motor skills, remains unclear.

The possibility that anxiety-related measures can be applied to a wide range of individuals with RTT does not necessarily mean that they are suitable from the psychometric viewpoint. The development of the RSBQ included a psychometric validation [24]; a recent re-evaluation expanded these analyses and demonstrated a high correlation between five of the RSBQ subscales that included the Fear/Anxiety subscale [16]. The latter actually correlated strongly with all other subscales with the exception of Walking/Standing, which suggests relative lack of specificity. Some efforts have also been made at delineating the psychometric properties of the ABC-C and ADAMS in groups of individuals with ID [30–32]; whether the latter data can be applied to RTT is unclear. For these reasons, we assessed the reliability and validity of the five anxiety-related subscales of the RSBQ, ADAMS, and ABC-C. Reliability was examined in three different ways: internal consistency, test-retest reliability, and intra-rater reliability. Cronbach’s alpha analyses showed that three of the subscales were in the good to excellent range, while the RSBQ Fear/Anxiety and the ABC Lethargy/Social Withdrawal were considered only fair. This finding contrasted with a less favorable assessment of the RSBQ Fear/Anxiety by Mount and colleagues (2002). Test-retest reliability was only performed on the RSBQ and ADAMS and demonstrated the three anxiety-related subscales were adequate, although the two ADAMS measures had better intraclass correlation coefficients. Intra-rater reliability analyses conducted on the same subscales showed a similar profile to test-retest reliability, with the ADAMS scales faring better than the RSBQ Fear/Anxiety, but all three were considered adequate. Altogether, these data indicate that the ADAMS anxiety subscales meet most criteria for adequate reliability. Unfortunately, we could not draw definitive conclusions about the ABC-C subscales since we were unable to evaluate its test-retest or intra-rater reliability.

Because of the lack of appropriate gold standards, validity of behavioral instruments is in general assessed by examining construct or concurrent validity. This means establishing that the measure is significant (and usually directly) correlated with other measures that evaluate the same construct (convergent validity), in this case anxiety. Complementing this, discriminant validity is demonstrated by poor correlations with measures of unrelated constructs [32]. These two types of validity can be thought of as sensitivity and specificity, respectively, and should be considered with caution as it is difficult to fully determine the relationship between behavioral constructs. Our data demonstrate that the three social anxiety subscales were strongly correlated among themselves and, among them, only the ADAMS Social Avoidance correlated with the two measures of general anxiety. On the other hand, the ADAMS General Anxiety and the RSBQ Fear/Anxiety were strongly correlated with each other. Although convergent validity was similar for general and social anxiety measures, the main difference was in terms of discriminant validity. While the general anxiety subscales correlated with measures of mood and other constructs such as hyperactivity, the social subscale correlations were restricted to mood and stereotypic behavior (excluding motor stereotypies). We can conclude that, in terms of concurrent validity, the ADAMS Social Avoidance, ABC-C Lethargy/Social Withdrawal, and ABC-C Social Avoidance are the best measures. Beyond psychometric properties, the value of behavioral instruments is also determined by their ability to measure clinically or functionally meaningful behaviors. This is a particularly important feature when selecting a behavioral instrument as an outcome measure in intervention studies. We used two complementary instruments to determine the functional implications of anxiety-related measures in RTT: adaptive behavior, a construct that evaluates cognition in practical aspects, and quality of life [46, 58]. Relationships between anxiety scales and adaptive behavior measures were weak; however, higher scores (i.e., more severe) on the ADAMS Social Avoidance were inversely correlated to a measure of psychosocial quality of life, indicating reduced psychosocial well-being.

It has been difficult to measure anxiety and mood abnormalities in individuals with ID. The best-established measure in the field, the ABC-C, emphasizes social type of anxiety although it also includes social indifferent behaviors more characterized in autism spectrum disorder [37, 38]. The ABC-C Social Avoidance, which has been derived from the ABC-C Lethargy/Social Withdrawal, represents an attempt at isolating more social anxiety-specific items of the ABC-C [38]. Consequently, the introduction of the ADAMS was an attempt at better delineating both social and non-social forms of anxiety in these populations [30, 31]. Our data suggest that, although all anxiety measures are adequate in RTT, social anxiety subscales and in particular the ADAMS Social Avoidance has the most solid psychometric foundation. Shortcomings of these anxiety measures seem to relate to their scoring structure, particularly the small number of items and the compressed range of scores. For instance, the RSBQ is scored on a three-point Likert scale (0–2) while the ADAMS and the ABC-C use a four-point Likert scale (0–3). The number of items included in a subscale is also important; the RSBQ Fear/Anxiety has only four items vs. the ADAMS General Anxiety subscale which has seven. However, content is also important; like the RSBQ Fear/Anxiety, the ABC-C Social Avoidance has only four items but exhibits a more favorable psychometric profile.

## Conclusions

This study represents the first attempt at systematically examining anxiety-like behavior in RTT using standardized instruments, concluding that social avoidance/anxiety is the most adequately measured anxious behavior. Anxiety-like behavior seems to be an important component of the RTT phenotype, with quality of life implications [12, 16]. Our reliance on informant-based behavior rating scales in RTT is an issue that cannot be underestimated. In addition to the aforementioned communication and motor deficits, abnormal muscle tone, and hand function impairments, psychomotor slowing (due to dyspraxia, etc.) is highly prevalent in RTT. This leads to difficulties in obtaining representative samples of behavior, unless observations are made for long periods of time in more naturalistic settings. This makes parents and caregivers critical informants in RTT and has led to other evaluation strategies such as home video assessments [59]. Our study had several limitations mentioned in the preceding sections. In addition to the inclusion of a number of subjects still undergoing regression and all subjects being under age 11, there was no comparison group with ID to determine the specificity of the anxious behavior profiles in RTT. We were also unable to complete reliability assessments of the ABC-C, and a number of our analyses had variable sample sizes. However, comparisons between subgroups with complete data with those with partial data did not show significant differences in anxiety-related measures. Additionally, the subjects’ regression status did not appear to influence their scores on anxiety scales. Nonetheless, the subject sample may have been biased since a large proportion of the participants were applicants to an ongoing phase 2 trial of IGF-1 in our institution. Although parents are not told the specific subscales and cutoff scores used to determine inclusion, parents are told that participants must demonstrate a “specific behavior profile based on standardized questionnaires.” Despite these and other possible shortcomings, the data reported here could serve as basis for the study of anxiety in RTT and for developing new assessment tools. The fact that anxiety-related scores were among the most responsive to IGF-1 in a recent trial [33] emphasizes the importance of defining an adequate method for measuring this behavioral abnormality. These findings elucidate the prevalence and severity of anxiety symptoms in RTT and lay the foundation for use of the aforementioned measures in future clinical trials.
